# Downregulation of early visual cortex excitability mediates oscillopsia suppression

**DOI:** 10.1212/WNL.0000000000004360

**Published:** 2017-09-12

**Authors:** Hena Ahmad, R. Edward Roberts, Mitesh Patel, Rhannon Lobo, Barry Seemungal, Qadeer Arshad, Adolfo Bronstein

**Affiliations:** From the Academic Department of Neuro-Otology, Charing Cross Hospital Campus, Imperial College London, UK.

## Abstract

**Objective:**

To identify in an observational study the neurophysiologic mechanisms that mediate adaptation to oscillopsia in patients with bilateral vestibular failure (BVF).

**Methods:**

We directly probe the hypothesis that adaptive changes that mediate oscillopsia suppression implicate the early visual-cortex (V1/V2). Accordingly, we investigated V1/V2 excitability using transcranial magnetic stimulation (TMS) in 12 avestibular patients and 12 healthy controls. Specifically, we assessed TMS-induced phosphene thresholds at baseline and cortical excitability changes while performing a visual motion adaptation paradigm during the following conditions: baseline measures (i.e., static), during visual motion (i.e., motion before adaptation), and during visual motion after 5 minutes of unidirectional visual motion adaptation (i.e., motion adapted).

**Results:**

Patients had significantly higher baseline phosphene thresholds, reflecting an underlying adaptive mechanism. Individual thresholds were correlated with oscillopsia symptom load. During the visual motion adaptation condition, no differences in excitability at baseline were observed, but during both the motion before adaptation and motion adapted conditions, we observed significantly attenuated cortical excitability in patients. Again, this attenuation in excitability was stronger in less symptomatic patients.

**Conclusions:**

Our findings provide neurophysiologic evidence that cortically mediated adaptive mechanisms in V1/V2 play a critical role in suppressing oscillopsia in patients with BVF.

Bilateral vestibular failure (BVF) is a collective term used to describe patients with total or subtotal loss of function of either the vestibular end organs or the vestibular cranial nerve. Because the vestibular-ocular reflex (VOR) provides gaze stabilization during head movements, avestibular patients lose this ability^1^ and experience an illusionary movement of the visual world, called head movement–induced oscillopsia.^2^

Patients report that over time the oscillopsia diminishes.^3–7^ Previous work has suggested that this compensatory process involves in part the generation of plastic oculomotor changes to improve gaze stability during head movements^3,6,8,9^ and perceptually mediated compensatory mechanisms such that avestibular patients become desensitized to visual motion.^5^ These aforementioned changes have additionally been shown to correlate with clinical outcome measures.^6,10^ Hence, gaining an understanding of the underlying neurophysiologic mechanisms that underpin adaptation to oscillopsia not only has a theoretical scientific importance but also is of critical clinical significance.^5,10–14^

To date, the neurophysiologic mechanisms that underpin centrally mediated adaptation to oscillopsia remain obscure. Previous neuroimaging findings have demonstrated that functional plasticity in the visual (V1/V2 and V5/MT) cortex contributes to adaptation after BVF.^15–18^ On the basis of the aforementioned findings, we hypothesize that neurophysiologic mechanisms associated with the early visual cortex mediate oscillopsia suppression. Accordingly, here we directly assessed visual cortical excitability in both vestibular patients and matched healthy controls using transcranial magnetic stimulation (TMS).

Given that avestibular patients have an absent VOR and thus are continuously exposed to retinal image slip, we predict on the basis of previous findings in normal individuals^19,20^ that (1) patients may have a more adapted (i.e., less excitable) visual cortex^19,20^ at rest (i.e., baseline threshold) compared to controls; (2) given that patients are less susceptible (i.e., already desensitized) to visual motion,^5^ implementation of a visual motion adaptation paradigm^20^ would have a less potent modulatory effect on cortical excitability in patients compared to healthy controls; and (3) if such findings are relevant to the central changes mediating oscillopsia adaptation, then they should relate to oscillopsia symptom load as measured with clinical questionnaires.

## METHODS

### Patient and clinical demographics.

Twelve right-handed^21^ patients with BVF were recruited from a tertiary referral clinic (Charing Cross Hospital) between November 2013 and December 2015 (7 men, age range 29–65 years; mean age 54.5 years, SD 11.9 years; table). All patients had a confirmed diagnosis for at least 1 year before participating in the experiment, and we ensured that there was no other neurologic disorder. Absent vestibular function was confirmed by a positive head impulse test bilaterally and a finely broken-up doll's eye movement (clinically), as well as absent VOR responses during bithermal caloric irrigations and constant-velocity rotations (90°/s) in the dark (oculography). Hearing was also formally assessed in all patients with a pure-tone audiogram and was found to be within normal limits for their age. Twelve right-handed matched healthy controls (7 men, age range 28–66 years, mean age 55 years, SD = 11.1 years) with no neurologic or audiovestibular disease were also recruited. All participants and healthy controls had normal or corrected normal visual acuity. No participants had any TMS contraindication.

**Table:**
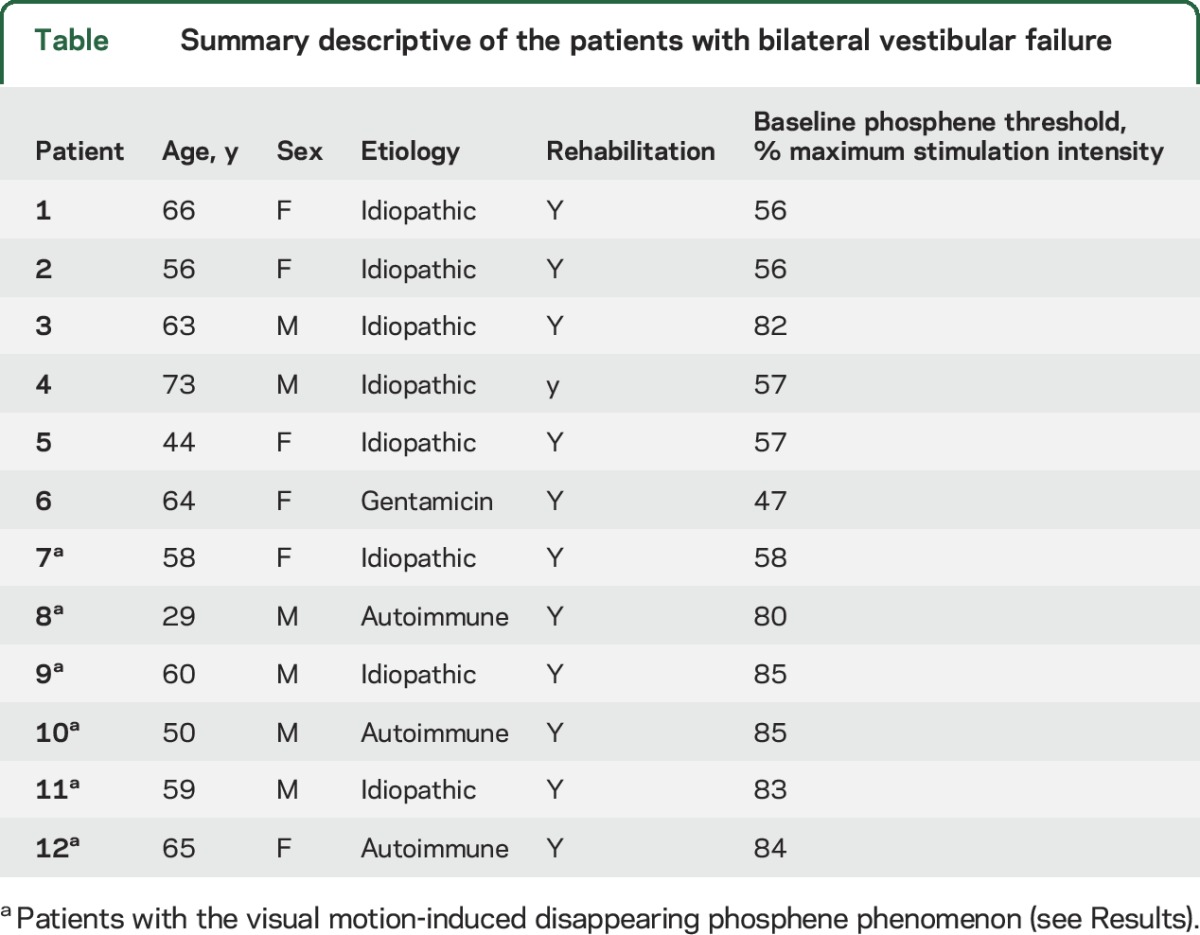
Summary descriptive of the patients with bilateral vestibular failure

### Standard protocol approvals, registrations, and patient consents.

All participants provided written informed consent in accordance with the requirements set out by the local ethics committee, which approved the study.

### Visual cortical assessment with TMS.

**Figure 1:**
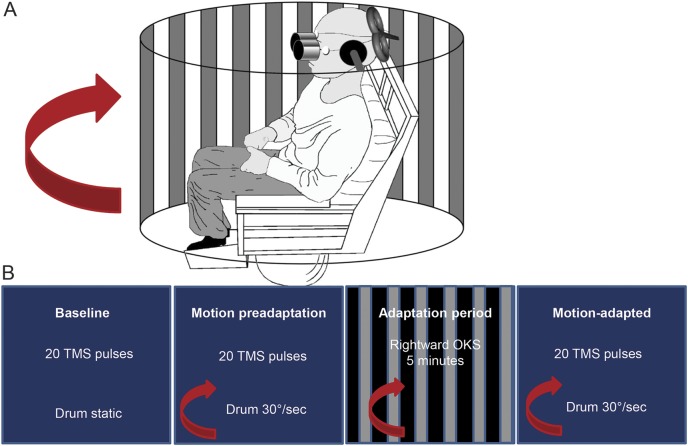
Experimental protocol (A) Experimental protocol used for the visual motion adaptation paradigm. Participants were seated in a stationary (locked) Barany chair surrounded by a full-field black and white curtain that was either stationary or rotated rightward (during motion conditions) at 30°/s. Eye movements were recorded with electro-oculography. Transcranial magnetic stimulation (TMS) was applied over the early visual cortex, and the curtain was viewed through the binoculars. The participant’s head was fixed with clamps over the ears. (B) Timeline of the experimental protocol. Initially, we established the individual phosphene threshold and then assessed baseline measures of cortical excitability by viewing the static curtain (i.e., baseline) with 20 TMS pulses followed by assessing cortical excitability with 20 TMS pulses during rightward motion of the curtain at 30°/s (i.e., motion preadaptation). We then adapted participants to rightward motion of the curtain at 30°/s for 5 minutes; immediately after this adaptation period, we assessed cortical excitability again during rightward visual motion with 20 TMS pulses (i.e., motion adapted). OKS = optokinetic stimulus.

TMS was used to assess V1/V2 excitability with perception-based phosphene measures. Phosphenes are illusory flashes of light that can be elicited via direct stimulation of the occipital cortex and reflect the underlying cortical excitability.^22–24^ We applied single biphasic TMS pulses (Magstim 200 stimulator, Magstim Co, Whitland, UK) using a 70-mm butterfly-shaped coil over V1/V2. The coil position was localized via a functional method as previously described.^25,26^ Initially, we placed the coil centrally over the inion with the coil handle turned laterally, and if required, we moved the coil dorsally in 1-cm incremental steps until a bright, stationary, midline phosphene was perceived by the participant.^19,20,27,28^ All participants were naive to the hypothesis but were told that the purpose of the experiment was to investigate changes in the visual part of the brain after the complete loss of inner ear balance function. All participants underwent a short standardized training session in darkness to familiarize them with phosphene detection.^23^ In this familiarization session, participants were informed that after the stimulus (i.e., TMS pulse) they may detect a phosphene and were asked to describe its shape, size, intensity, and location on an imaginary clock face,^28^ the last additionally monitoring accurate coil position. After localization, the coil was secured in place with rigid clamps for the remainder of the experiment. The participant’s head was also secured with both a chin rest and foam ear pads (figure 1).

### Establishing TMS visual cortical thresholds.

We established TMS thresholds using a modified binary search paradigm that allowed us to determine the TMS intensity required to elicit a phosphene 50% of the time during a trial sequence. This paradigm is an adaptive procedure in which an initial TMS pulse is given at a value representing the bisection of an initial upper and lower boundary pair. These boundaries are continually updated on the basis of the participant's prior response to each TMS pulse (i.e., a positive subjective response shifts the boundary downward, whereas a negative response shifts the boundary upward). Determination of the threshold is made when the participant makes 3 consecutive alternative choices. Once determined, we proceed to ascertain its accuracy by applying 20 TMS pulses, each separated by 6 seconds. In response to each pulse, participants were required to provide a binary response (i.e., either yes or no) to whether they perceived a phosphene. If the established threshold was correct, we observed between 8 and 12 yes responses. If the responses fell outside this range, the threshold was re-established.^20,28^ (In the present study we did not asses late visual cortex excitability [V5/MT] because of the associated difficulties of perceiving moving phosphenes during visual motion.)

### Experimental protocol.

Both threshold detection (above) and the visual motion adaptation experiment (figure 1) took place in dim lighting with eyes open. Participants were seated and surrounded by an 1.44-m-diameter drum that consisted of black and white vertical stripes (i.e., optokinetic stimulus) at 0.1 cycle per degree viewed at a fixed distance of 0.72 m (subtending a 30° field of view), as depicted in figure 1.^20^ The drum was either stationary during baseline or moving rightward at 30°/s during both the preadaptation and motion adapted conditions. We used only rightward motion because previous work has demonstrated no directional differences of motion on cortical excitability.^20^ Participants viewed the drum through a pair of goggles fixed to the chair to restrict peripheral visual field and hence illusory sensations of self-motion called vection (googles were removed during the 5-minute adaptation phase).^20^ To further ensure that participants did not develop vection during visual motion viewing, participants planted their feet on the solid ground to reassure them that they were stationary.^29^ Eye movements were recorded with horizontal electro-oculography (figure 1).

### Questionnaires.

All patients completed a validated oscillopsia scale questionnaire^10^ to assess the functional status and disability secondary to oscillopsia. The scale required modification for 1 patient with orthopedic comorbidities that interfered with the mobility part of the questionnaire. The modification to the questionnaire for this patient was performed blindly by a consultant neurologist involved in the design of the original oscillopsia questionnaire (A.B.).

### Data analysis.

Results were analyzed offline by calculating the probability of phosphene perception. For example, if a participant’s 50% baseline threshold [TMS intensity producing *p* (λ) = 0.5 at baseline, i.e., 10 of 20 yes responses] increased to *p* (λ) = 0.7 (14 of 20 yes responses), that would reflect a 20% increase in visual cortical excitability. Statistical analysis was performed with SPSS 22 (SPSS, Inc, Chicago, IL).

## RESULTS

**Figure 2:**
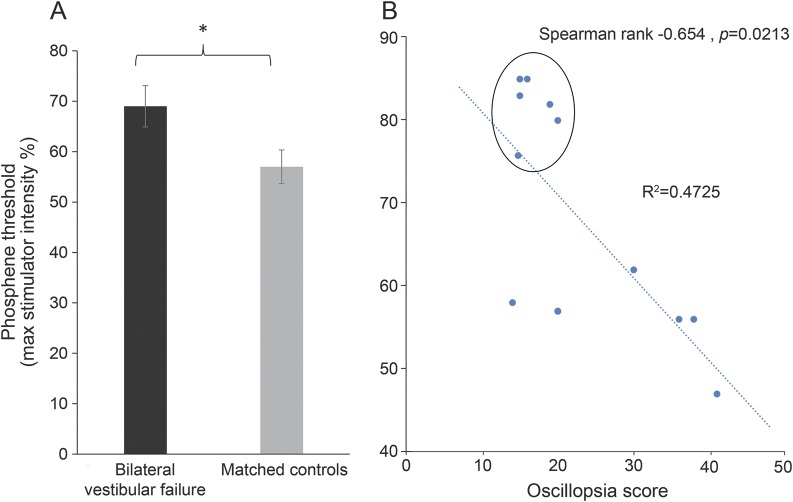
Baseline cortical excitability (A) Baseline phosphene thresholds in patients with bilateral vestibular failure (BVF; black bar) or healthy controls (gray bar) presented in percent of the maximum transcranial magnetic stimulation (TMS) stimulator output. As shown, patients had significantly higher baseline thresholds, i.e., needed higher-intensity TMS to elicit a phosphene, compared to healthy controls. Error bars denote standard error. *Significant at *p* < 0.05. (B) Relationship between oscillopsia symptom score as assessed with the validated questionnaire^10^ on the x-axis and the baseline phosphene thresholds presented in percent of the maximum stimulator output on the y-axis. Higher oscillopsia scores were associated with lower phosphene thresholds. The circled data points belong to the patients with BVF with the visual motion-induced disappearing phosphene phenomenon (see Results).

During establishment of baseline phosphene thresholds, we observed that patients with BVF required a higher level of maximum stimulator output to reach threshold compared to healthy controls. In patients, the stimulator intensity required to reach threshold was 69% of the maximum output (SD = 14.2) compared to 57% in healthy controls (SD = 11.6) (*p* = 0.02, independent-samples *t* test; figure 2A). In the patient group, we observed a relationship between oscillopsia symptom load and baseline TMS thresholds in that those patients with high thresholds (i.e., low V1/V2 excitability) were found to be symptomatically less troubled by oscillopsia compared to those patients with higher visual cortical excitability (i.e., lower thresholds; *R*^2^ = −0.654, *p* = 0.02; figure 2B).

**Figure 3:**
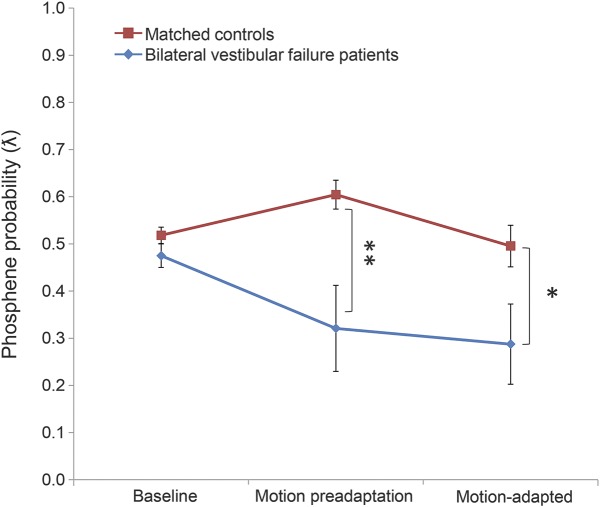
Changes in cortical excitability during and after visual motion Results from the visual motion adaptation paradigm. On the x-axis are the different measurement conditions, namely baseline, motion preadapted, and motion adapted; the probability of perceiving a phosphene is shown on the y-axis. Red line reflects the trend observed in healthy controls; blue line reflects the trend observed in patients. There were no differences in the probability of perceiving a phosphene at baseline between healthy controls and avestibular patients, but there was a significantly lower probability of perceiving a phosphene during both the motion preadapted and motion adapted conditions in avestibular patients compared to healthy controls. Error bars denote standard error.

Having established the TMS (50%) thresholds, we now turn to the results of the visual motion adaptation paradigm. As expected, we observed no differences between patients and healthy controls during baseline measures, indicating that the thresholds previously obtained were consistent (*p* > 0.05, *t* test; figure 3). We then proceeded to investigate the effects on visual cortical excitability during motion and after visual motion adaptation. We observed that in patients the probability of phosphene perception decreased during both preadaptation and postadapted conditions compared to baseline (static) measures. Conversely, in the healthy controls, we observed that during preadaptation, the probability of perceiving a phosphene increased, whereas after motion adaptation, the probability of perceiving a phosphene decreased (i.e., postadapted; figure 3).

We performed a 2 × 3 analysis of variance to examine the variation of phosphene perception with 1 within-subject factor (motion condition, 3 levels: baseline, motion preadaptation, and motion adapted) and 1 between-subject factor (group: healthy controls vs avestibular patients). We observed a main effect of group (*F*_1,11_ = 8.16, *p* = 0.009) and condition (*F*_2,22_ = 3.23, *p* = 0.049). There was also a condition × group interaction (*F*_2,22_ = 4.53, *p* = 0.016). Post hoc *t* tests revealed no difference between the groups during baseline (patients: mean = 0.48, SD = 0.09; healthy controls: mean = 0.52, SD = 0.06, *p* = 0.18); however, there was a difference between patients and healthy controls during both motion preadaptation (patients: mean = 0.32, SD = 0.32; healthy controls: mean = 0.61, SD = 0.10, *p* = 0.009) and motion adapted (patients: mean = 0.29, SD = 0.29; healthy controls: mean = 0.50, SD = 0.14, *p* = 0.043) conditions (figure 3).

**Figure 4:**
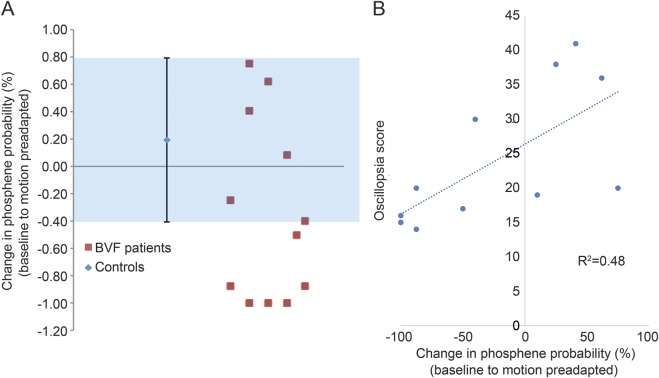
Changes in cortical excitability from baseline to motion (A) The y-axis shows the percent change in the probability of perceiving a phosphene from baseline to the motion preadapted condition. Blue shaded areas represent the healthy controls with an error bar of 3 SDs. The change for each patient is represented by the red squares. Patients with disappearing phosphenes during visual motion onset fell outside the 3 SDs. (B) The relationship between the percent change in the probability of perceiving a phosphene from the baseline to the motion preadapted condition on the x-axis and the oscillopsia symptom score on the y-axis. BVF = bilateral vestibular failure.

Given the drop in the phosphene perception in patients with BVF during visual motion onset, we proceeded to inspect the data more closely. We identified a subset of avestibular patients in whom it was not possible to elicit phosphenes during visual motion (i.e., the preadaptation and motion adapted conditions) despite the fact that in the baseline condition there was no difference in the ability to perceive phosphenes. Thus, we probed this motion-induced “disappearing phosphene” phenomenon further by calculating each participant's percentage change in phosphene perception (probability of yes/no responses) from the baseline to the preadaptation condition to assess the distribution of patient responses. We identified 6 patients who fell outside 3 SDs of the mean of the rest of the patients (n = 6) and all the controls (figure 4A). Furthermore, we investigated the relationship between motion-induced percentage change in phosphene perception and oscillopsia scores and observed that patients with disappearing phosphenes during motion had lower symptom scores compared to patients with persisting phosphenes during visual motion (*R*^2^ = 0.48, *p* < 0.01 Pearson correlation; figure 4B).

Finally, the mean slow-phase velocity of the optokinetic nystagmus was 26°/s. No relationship between optokinetic slow-phase velocity and phosphene perception was observed in any of the experimental conditions (*p* > 0.05), as previously reported in normal controls.^20^

## DISCUSSION

In this study, we probed the neurophysiologic mechanisms that underpin adaptation (subjective improvement) to oscillopsia in avestibular patients. We observed that patients with BVF have a less excitable early visual cortex at baseline compared to healthy controls. Moreover, we observed that individual baseline cortical thresholds (V1/V2) were found to be correlated with the degree of functional disability associated with the oscillopsia. That is, less symptomatic (i.e., better adapted) patients had a less excitable visual cortex compared to more functionally impaired patients (i.e., poorly adapted, higher cortical excitability). This initial finding suggests that everyday exposure to excessive retinal slip initiates a cortically mediated compensatory process, resulting in downregulation of V1/V2 excitability, similar to that observed in healthy controls after prolonged visual motion adaptation^19,20^ and during involuntary eye oscillations.^30^ Given that such downregulation of excitability correlates with the oscillopsia questionnaire scores, the findings indicate that background visuo-cortical excitability levels may partly mediate clinical recovery.

We then proceeded to examine the effects of a visual motion adaptation paradigm on V1/V2 excitability before and after adaptation. During visual motion (before adaptation), we observed a significant decrease in phosphene perception in patients, whereas contrastingly, we observed an increase in healthy controls. This finding in healthy controls is in agreement with our previous findings that we attributable to a nonspecific effect (i.e., generalized arousal or attention) in response to visual motion.^19,20^ Further group differences in cortical excitability between patients and healthy controls were found after adaptation. In healthy controls, cortical excitability was reduced, which again is in line with our previous reports in young healthy controls,^20^ whereas in patients we observed no differences in cortical excitability when comparing the preadaptation and motion adapted conditions. Hence, our results illustrate a dissociation in behavior when comparing participants with and without vestibular function. We postulate that the dissociations observed may be attributable to the 2 following non–mutually exclusive explanations: there is a preexisting cortical adaptation induced by the constant retinal image slippage and oscillopsia in patients,^19,20^ and avestibular patients are desensitized to the influence of further visual motion,^5,13^ i.e., a ceiling effect.

As mentioned, post hoc inspection of the patient data revealed a subset of patients who did not perceive phosphenes during visual motion (disappearing phosphenes). To confirm that this was not attributable to a technical artifact (i.e., coil or head movement), we recalled 2 patients from this subgroup and repeated the experiment. Once again, we observed the rapid disappearance of the phosphenes during visual motion within 10 to 15 seconds to values <5% of those observed in the first experimental session. Moreover, in these 2 patients, we further increased the stimulator intensity output by 10% above their individual stimulator thresholds (close to the maximum possible stimulator output) during visual motion, but this did not allow the visualization of phosphenes. Ten seconds after the motion had ceased, both patients could once again perceive phosphenes at threshold. Notably, these 6 patients exhibited a unique characteristic (figure 1B) in that they had significantly higher baseline thresholds of the early visual cortex compared to those patients with persisting phosphenes and were less functionally impaired by the oscillopsia.

Accordingly, centrally mediated oscillopsia adaptation after BVF may occur by downregulation of V1/V2 cortical excitability. Moreover, we speculate that it is possible that a similar cortical mechanism may be active for the reduction of oscillopsia secondary to excessive eye movements such as congenital nystagmus and nystagmus secondary to brainstem disease.^30–32^ In addition, more marked suppression of cortical excitability is associated with enhanced functional recovery from oscillopsia and reduced cortical responsiveness to visual motion. Accordingly, patients with oscillopsia may benefit from enhanced rehabilitation with repeated visual motion paradigms to reduce cortical excitability, which in turn improves functional outcome. Our results may have potential future clinical implications for patient treatment via implementation of pharmacologic or electric neuromodulation of visuo-cortical excitability levels.

## Supplementary Material

Accompanying Editorial

## GLOSSARY

BVF: bilateral vestibular failure
TMS: transcranial magnetic stimulation
VOR: vestibular-ocular reflex
