# Supplementary material for: Downregulation of early visual cortex excitability mediates oscillopsia suppression
Source: Neurology. 2017 Sep 12;89(11):1179–85. doi: 10.1212/WNL.0000000000004360 (PMC5595274; doi:10.1212/WNL.0000000000004360)
Supplement: Accompanying Editorial [file supp_89_11_1179__index.html]

Downregulation of early visual cortex excitability mediates oscillopsia suppression — Accompanying Editorial 

# Downregulation of early visual cortex excitability mediates oscillopsia suppression

## Accompanying Editorial

**Neurology® data supplements are not copyedited before publication. Published editorials and translations have been copyedited.  
 © 2017 American Academy of Neurology.  
  
 Files in this Data Supplement:**

- Accompanying Editorial - PDF
