# Supplementary material for: Downregulation of early visual cortex excitability mediates oscillopsia suppression
Source: Neurology. 2017 Sep 12;89(11):1179–85. doi: 10.1212/WNL.0000000000004360 (PMC5595274; doi:10.1212/WNL.0000000000004360)
Supplement: Accompanying Editorial [file supp_WNL.0000000000004360_WNL.0000000000004368.pdf]

As the world turns, why do some people adapt to vestibular failure and others do not?

Steven L. Galetta, MD

Correspondence to Dr. Galetta: steven.galetta@nyumc.org

Neurology® 2017;89:1–2

Chronic vertigo and oscillopsia are 2 of the most vexing problems that neurologists encounter. Even when the etiology is known, there is often a limit to the therapy that can be offered to the patient beyond vestibular rehabilitation. Oscillopsia diminishes over time in many individuals, but the mechanism for this adaptive change is unclear. In this issue of *Neurology*®, Ahmad et al.[1] examine the central mechanisms that may be responsible for adaptation after bilateral vestibular failure. Most of the cases in this cohort had vestibular failure from either idiopathic or autoimmune causes. Ahmad et al. found that the patients with bilateral vestibular failure had reduced visual cortical excitability compared to controls under both static and motion conditions.

Classically, compensatory mechanisms for oscillopsia result from either ocular motor changes to improve gaze stabilization or central mechanisms that dampen the excitability of the brain in response to motion stimuli.[2,3] Studying 12 patients with impaired oculocephalic and caloric irrigation responses, but with normal hearing and no other neurologic abnormalities, the authors compared this cohort to healthy controls at baseline (static condition), as they became exposed to visual motion, and after visual adaptation to visual motion. They measured the perception of transcranial magnetic stimulation (TMS)–induced phosphenes, generated by TMS applied over the visual cortex, in all conditions. The stimulator intensity to induce phosphenes and the likelihood of phosphene elicitation provided a readout of visual cortical excitability. The authors found that patients with bilateral vestibular failure had higher phosphene thresholds compared to healthy controls, showing an overall diminished excitability in the V1/V2 areas of these patients. The likelihood of eliciting phosphenes further decreased during and after motion adaptation in these patients, showing that their visual cortical excitability diminished in response to visual motion stimuli. In contrast, healthy controls had an increase in cortical excitability during motion preadaptation, perhaps reflecting either increased attention or arousal, and a decrease in excitability after motion adaptation. In all testing conditions, patients with the least amount of functional disability also had the lowest V1/V2 excitability (i.e., highest phosphene thresholds or lowest likelihood of eliciting phosphenes). These findings collectively suggest that decreased visual cortical excitability, both at rest and in response to visual motion, is associated with functional adaptation to oscillopsia.

The import of this article is that it provides a potential explanation as to why certain patients fare reasonably well after vestibular injury while others struggle to compensate. The study does not establish visual cortical excitability changes as the cause of the functional adaptation to oscillopsia. However, the findings suggest that neuromodulatory approaches to diminish visual cortical excitability could be incorporated into trials for functional restoration in poorly recovered patients. In addition, the paradigm could be used to examine the visual adaptation of patients with congenital nystagmus and other forms of acquired nystagmus.

The big question is whether visual cortical excitability, as assayed by TMS-induced phosphene thresholds, is a neurophysiologic marker of adaptation to vestibular failure. The ability to perceive motion involves a complex network of centers beyond the primary visual cortex and into the higher association cortex, including V5/MT. We do not fully understand the complex interactions of these networks and how they may be influenced by other inputs and outputs. Recovery of visual loss from optic neuritis and other visual disorders may be influenced by the cortical reorganization and plasticity of the visual cortex.[4,5] In a similar fashion, neuroplasticity in V1/V2 and V5/MT occurs with adaptation in motion tasks in healthy persons and patients with vestibular dysfunction, and each cortical area may be differentially affected.[6,7] Thus, it has yet to be determined whether the diminished V1/V2 excitability seen in the present study indicates that these areas are the primary drivers of functional adaptation or if this excitability is an epiphenomenon of a network change underlying functional adaptation.

The authors also did not specifically obtain thresholds for moving phosphenes, which would provide information about V5/MT excitability, because patients had difficulties detecting them while simultaneously

See page 1179

From the Department of Neurology, NYU Langone, New York, NY.

Go to Neurology.org for full disclosures. Funding information and disclosures deemed relevant by the authors, if any, are provided at the end of the editorial.

© 2017 American Academy of Neurology

© 2017 American Academy of Neurology. Unauthorized reproduction of this article is prohibited.

experiencing visual motion. So, the response of V5/MT excitability in patients undergoing motion adaptation was not specifically examined in this study. In these patients, a multimodal research approach could be considered: TMS assays to delineate excitability change in cortical centers that process motion detection, clinical assays to document degree of oscillopsia, and functional assays to gauge the ill effects of abnormal motion perception. Perturbation with neuromodulatory or rehabilitative interventions can further establish causality. Future studies could also focus on other potential factors associated with oscillopsia suppression, including the extent of injury, the duration of the dysfunction, the asymmetry of the vestibular involvement, the type of vestibular dysfunction, and the interaction of other eye movement systems, including the use of the saccade system to compensate for the retinal slip that may occur with head movements. Collectively, these investigations could generate new directions for the treatment of oscillopsia, providing hope to those who have a shaky view of their visual world.

STUDY FUNDING

No targeted funding reported.

DISCLOSURE

Dr. Galetta has received consulting honorarium from Biogen. Go to Neurology.org for full disclosures.

REFERENCES

1. Ahmad H, Roberts RE, Patel M, et al. Downregulation of early visual cortex excitability mediates oscillopsia suppression. Neurology 2017;89:1179–1185.
2. Gresty MA, Hess K, Leech J. Disorders of the vestibulo-ocular reflex producing oscillopsia and mechanisms compensating for loss of labyrinthine function. Brain 1977;100:693–716.
3. Bronstein AM, Hood JD. Oscillopsia of peripheral vestibular origin: central and cervical compensatory mechanisms. Acta Otolaryngol 1987;104:307–314.
4. Jenkins TM, Toosy AT, Ciccarelli O, et al. Neuroplasticity predicts outcome of optic neuritis independent of tissue damage. Ann Neurol 2010;67:99–113.
5. Martins Rosa A, Silva MF, Ferreira S, et al. Plasticity in the human visual cortex: an ophthalmology-based perspective. Biomed Res Int 2013;2013:568354.
6. Lubeck AJ, Van Ombergen A, Ahmad H, et al. Differential effect of visual motion adaptation upon visual cortical excitability. J Neurophysiol 2017;117:903–909.
7. Deutschlander A, Hufner K, Kalla R, et al. Unilateral vestibular failure suppresses cortical visual motion processing. Brain 2008;131:1025–1034.

© 2017 American Academy of Neurology. Unauthorized reproduction of this article is prohibited.
